# Inhibition of autophagy promotes ultrasound‑targeted microbubble destruction-induced apoptosis of pancreatic cancer cells

**DOI:** 10.7150/ijms.106509

**Published:** 2025-03-03

**Authors:** Nan Chen, Xiaoyu Zhang, Ping Yang, Xuemei He

**Affiliations:** 1 Department of Ultrasound, The First Affiliated Hospital of Chongqing Medical University, Chongqing 400016, China.; 2 Department of Urology, The First Affiliated Hospital of Chongqing Medical University, Chongqing 400016, China.

**Keywords:** ultrasound-targeted microbubble destruction (UTMD), chloroquine (CQ), apoptosis

## Abstract

In therapeutic studies of pancreatic cancer, ultrasound-targeted microbubble destruction (UTMD) has shown potential in promoting apoptosis as a safe and non-invasive adjuvant therapy. Autophagy, a regulatory mechanism for cellular stress response and survival, plays a dual role in tumor development, progression, and treatment. However, the role of autophagy in UTMD-induced apoptosis in pancreatic cancer cells remains unclear. In this study, chloroquine (CQ), an autophagy inhibitor, was combined with UTMD to treat pancreatic cancer both *in vitro* and* in vivo*, with changes in apoptosis assessed through Western blot and TUNEL staining. The results showed that UTMD induced both apoptosis and autophagy in pancreatic cancer cells. Notably, inhibiting autophagy significantly enhanced UTMD-induced apoptosis, while the inhibition of apoptosis did not affect UTMD-induced autophagy. These findings suggest that autophagy reduces the effectiveness of UTMD in treating pancreatic cancer. This study offers a new perspective on UTMD for treating pancreatic cancer, suggesting that combining autophagy inhibitors could be a promising strategy to enhance the effectiveness of pancreatic cancer therapy.

## 1. Introduction

Pancreatic cancer is the third leading cause of cancer-related deaths, with a five-year survival rate of only 13% 1, and its incidence and mortality rates continue to rise annually 2. Early diagnosis and treatment of pancreatic cancer remain major challenges 3. Although treatments such as surgical resection, radiotherapy, and chemotherapy have improved the survival rate of pancreatic cancer patients to some extent, these therapies are limited by low selectivity, systemic toxicity, and drug resistance 4-6. Therefore, continuous efforts are needed to explore new treatments that could improve the efficacy of pancreatic cancer therapy.

Ultrasound-targeted microbubble destruction (UTMD) is a non-invasive treatment that utilizes cavitation generated by the expansion and rupture of microbubbles (MBs) under ultrasound (US) to achieve therapeutic outcomes 7. Several studies have demonstrated that US with MBs induces apoptosis in colon, breast, and pancreatic cancer cells, while also inhibiting tumor invasion in pancreatic cancer cells 8-10. However, the mechanisms by which UTMD treatment mediates cell death remain unclear.

Autophagy is a highly conserved metabolic degradation process, a physiological mechanism that maintains cellular homeostasis by removing damaged or excess cellular components under stress conditions 11. Autophagy facilitates the recirculation of intracellular compounds. A portion of the cellular cytoplasm, including organelles, is engulfed and enters vesicles, which fuse with lysosomes and are degraded to produce new metabolites that fuel cellular metabolism and energy pathways 12. Previous studies have shown that UTMD treatment often leads to changes in cellular autophagy 13-15, with a close link between autophagic and apoptotic pathways 16. Nevertheless, the functional significance of autophagy-mediated alterations by UTMD in the regulation of pancreatic cancer cell apoptosis has not been fully elucidated.

**Figure 1:**
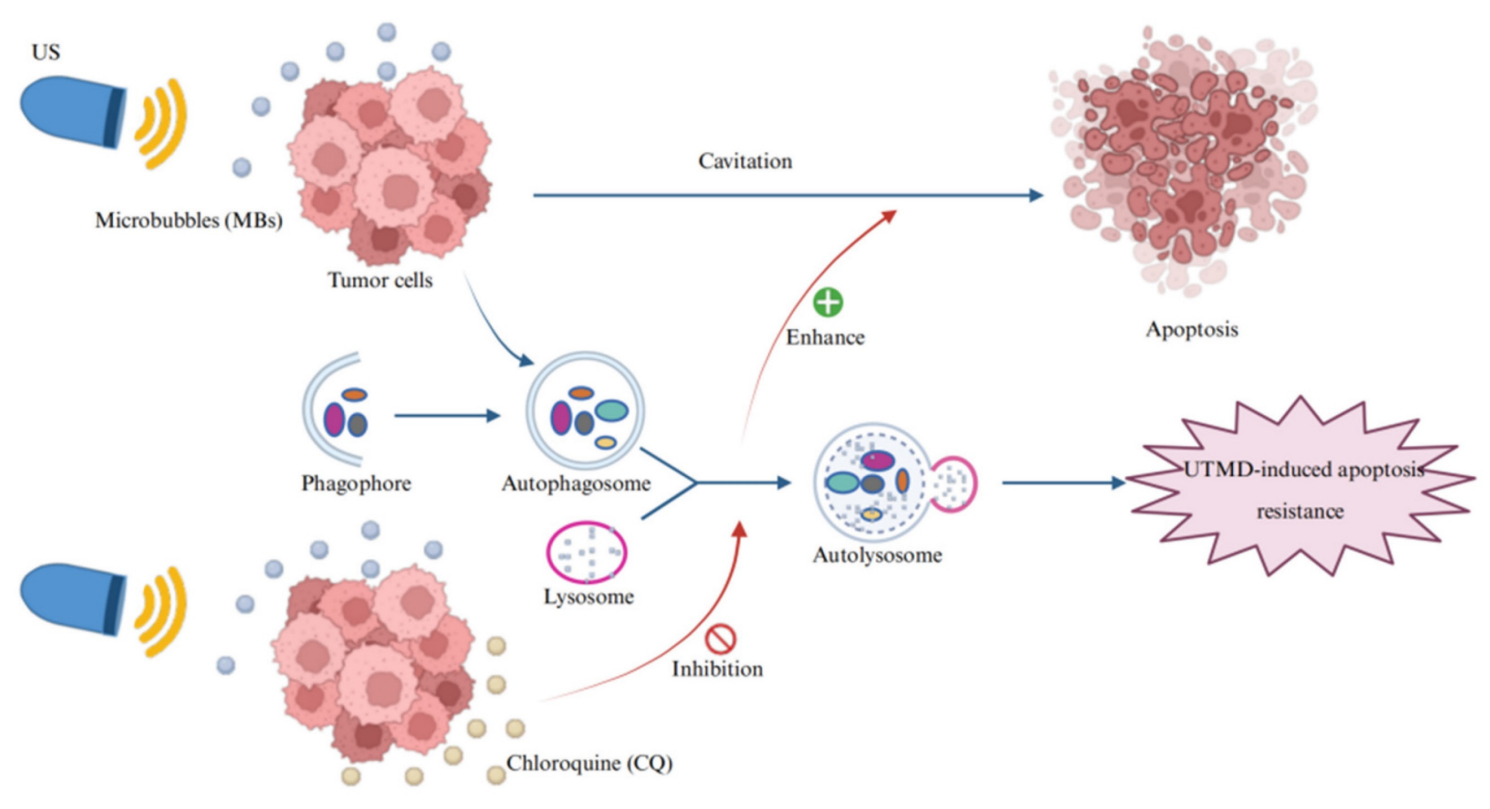
Schematic illustration of the combination therapy of UTMD with CQ. CQ inhibits UTMD-induced autophagy in pancreatic cancer cells, thereby promoting UTMD-induced apoptosis in these cells.

Therefore, this study aimed to investigate the dual effects of UTMD on both apoptosis and autophagy, as well as the regulatory role of autophagy in UTMD-induced apoptosis in pancreatic cancer cells. These investigations may provide a novel non-invasive therapeutic strategy for pancreatic cancer treatment (Figure 1).

## 2. Materials and Methods

### 2.1. Ultrasonic Equipment and Microbubbles (MBs)

The low-intensity focused ultrasound experimental device (Nercum, Chongqing, China) was equipped with a therapeutic transducer having an ultrasound output frequency of 650 KHz ± 10%, a focal length of 28 mm ± 15%, a duty cycle of 30%, and adjustable ultrasound intensity. The echocontrast agent SonoVue (Bracco, Milan, Italy) was used as the MBs to enhance cavitation. It was suspended in 5 mL of saline and thoroughly shaken before use. The MB/cell ratio was maintained at approximately 100:1, as recommended by Pan *et al.* 17. Cell suspensions or tumors were exposed to ultrasound for 5 minutes.

### 2.2. Cell culture and processing

Human pancreatic cancer cell lines PANC-1 and BXPC-3 were purchased from Procell (Wuhan, China). PANC-1 cells were cultured in DMEM, while BXPC-3 cells were cultured in RPMI-1640, both supplemented with 10% fetal bovine serum (Procell, Wuhan, China; Cat. No. 164210) and maintained at 37°C in a 5% CO_2_ atmosphere. According to the experimental protocol, the autophagy inhibitor CQ (Sigma-Aldrich, St. Louis, MO, USA; Cat. No. C6628) was added to the medium at a final concentration of 20 µmol/L or the apoptosis inhibitor Z-VAD-FMK (APExBIO, Houston, TX, USA; Cat. No. A1902) was added at a final concentration of 10 µmol/L. All experiments were performed when cells reached 80-90% confluence. After UTMD treatment, each group was incubated at 37°C for either 24 or 48 hours in a 5% CO₂ incubator for subsequent experimental tests.

### 2.3. Screening of ultrasound intensity

To investigate the effects of different UTMD intensities on pancreatic cancer cell apoptosis, we divided the cells into four groups based on ultrasound intensity: 1.0 W/cm^2^, 2.0 W/cm^2^, 3.0 W/cm^2^, and a control group. Pancreatic cancer cells in each group were treated with UTMD for 5 minutes, followed by 24 hours of incubation. The above experiments were repeated, and the cells were cultured for 48 hours after UTMD treatment. The apoptosis rate of pancreatic cancer cells in each group was analyzed by flow cytometry at different incubation times, and the ultrasound intensity that resulted in the highest apoptosis rate was selected.

### 2.4. The effect of UTMD on apoptosis and autophagy and the selection of appropriate incubation time

To further explore the effect of incubation time on UTMD-induced apoptosis and autophagy, pancreatic cancer cells treated with UTMD were incubated separately for 24 and 48 hours, using the ultrasound intensity selected in the above experiments. Multiple assays were employed to assess apoptosis and autophagy, and the incubation time that resulted in the most significant changes in both processes was identified.

### 2.5. Effect of autophagy on UTMD-induced apoptosis

Using the ultrasound intensity and incubation time determined in the above experiments, we further explored whether autophagy affects UTMD-induced apoptosis in pancreatic cancer cells. We compared the apoptosis among the control, US, CQ, UTMD, and UTMD combined with CQ groups using TUNEL staining and Western blot analysis.

### 2.6. Effect of apoptosis on UTMD-induced autophagy

Using the ultrasound intensity and incubation time determined in the above experiments, we further investigated whether apoptosis affects UTMD-induced autophagy in pancreatic cancer cells. We compared the apoptosis rates and autophagy levels among the control, UTMD, Z-VAD-FMK, and UTMD combined with Z-VAD-FMK groups using flow cytometry and Western blot analysis.

### 2.7. Flow cytometry

The cells to be examined were collected, rinsed twice with PBS, and then resuspended in 500 µL of PBS. The cells were transferred into 1.5 mL EP tubes and stained using the Annexin V-fluorescein isothiocyanate (FITC)/PI assay according to the manufacturer's protocol (Beyotime, Shanghai, China; Cat. No. C1062). Apoptosis was detected by flow cytometric analysis, and data were analyzed using Cytexpert V2.3 software (Beckman Coulter, Inc., Brea, CA, USA). The total apoptotic rate was quantified as the cumulative percentage of both early and late apoptotic cell populations.

### 2.8. Western blot analysis

Total protein was extracted using RIPA Lysis Buffer (Meilunbio, Dalian, China). Sodium dodecyl sulfate polyacrylamide gel electrophoresis (SDS-PAGE) was used for protein separation, followed by the transfer of proteins to nitrocellulose membranes (membranes were cut horizontally). The membranes were then incubated with primary antibodies, including anti-ACTB (Sangon Biotech, Shanghai, China; Cat. No. D110001), anti-Bcl-2 (HuaBio, Hangzhou, China; Cat. No. ET1702-53), anti-PARP (Proteintech, Rosemont, IL, USA; Cat. No. 13371-1-A), anti-Bax (Proteintech, Rosemont, IL, USA; Cat. No. 50599-2-Ig), anti-Cleaved Caspase-3 (Cell Signaling Technology, Danvers, MA, USA; Cat. No. 5A1E), anti-Beclin1 (Proteintech, Rosemont, IL, USA; Cat. No. 11306-1-AP), anti-p62/SQSTM1 (HuaBio, Zhejiang, China; Cat. No. HA721171), and anti-LC3B (Abcam, Cambridge, UK; Cat. No. ab192890). Images were analyzed using ImageJ (v1.54f).

### 2.9. Ad-mCherry-GFP-LC3 assay

Ad-mCherry-GFP-LC3 was transfected into pancreatic cancer cells for 48 hours to assess autophagic flux. Briefly, 1 × 10^5^ pancreatic cancer cells were grown in a 24-well plate and infected with lentivirus for 48 hours. The cells were then treated with UTMD, and after an additional 48 hours, all samples were examined using a Confocal Laser Scanning Microscope equipped with a 63× oil objective.

### 2.10. TUNEL staining

Terminal deoxynucleotidyl transferase dUTP nick end labeling (TUNEL) staining was performed using a One-step TUNEL In Situ Apoptosis Kit (Green, Elab Fluor® 488, Elabscience, E-CK-A321) to detect tumor apoptosis, following the manufacturer's instructions. Microscopic images were analyzed using ImageJ (v1.54f).

### 2.11. Mouse xenograft assays

BXPC-3 cells (3 × 10^6^) were injected into the flanks of 5-week-old male Nu/Nu nude mice (Beijing Vital River Laboratory Animal Technology Co., Ltd., Beijing, China). After one week of feeding, 35 tumor-bearing mice were collected and evenly divided into five groups: Control, US, CQ, UTMD, UTMD+CQ. Except for the control and CQ groups, the other three groups were treated with UTMD at the previously determined intensity for 5 minutes. The nude mice in the Control, US, and UTMD groups were intraperitoneally injected with 0.9% saline, while the mice in the CQ and UTMD+CQ groups were intraperitoneally injected with CQ (60 mg/kg). Treatments were administered every three days, and tumor volume (calculated using the formula: length (L) × width (W)^2^ × 0.5, where L is the longest diameter and W is the shortest diameter) and body weight were measured before each treatment. The nude mice were euthanized at the end of the study, and *in vivo* solid tumors were dissected for analysis. Fresh tumor tissues were fixed in 4% paraformaldehyde, embedded in paraffin, and sectioned into 3 μm slices. The sections were then deparaffinized, rehydrated, after which apoptosis was assessed using TUNEL staining.

The animal experiments complied with the Animal Research: Reporting of *In Vivo* Experiments (ARRIVE) guidelines. The study was carried out in accordance with the Guidance on the Operation of the Animals (Scientific Procedures) Act 1986 and associated guidelines. The animal study protocol was approved by the Institutional Animal Care and Use Committee (IACUC) of Chongqing Medical University (IACUC-CQMU-2024-11117). All animal care and handling procedures adhered to the IACUC guidelines.

### 2.12. Statistical analysis

All experiments were independently repeated at least three times. The data were analyzed using GraphPad Prism 8.0 software and presented as the mean ± standard deviation (SD). A t-test was performed to compare differences between two groups, while a one-way analysis of variance (ANOVA) was used to analyze differences among multiple groups. Statistical significance was defined at p < 0.05.

## 3. Results

### 3.1. Ultrasound parameter screening result

**Figure 2:**
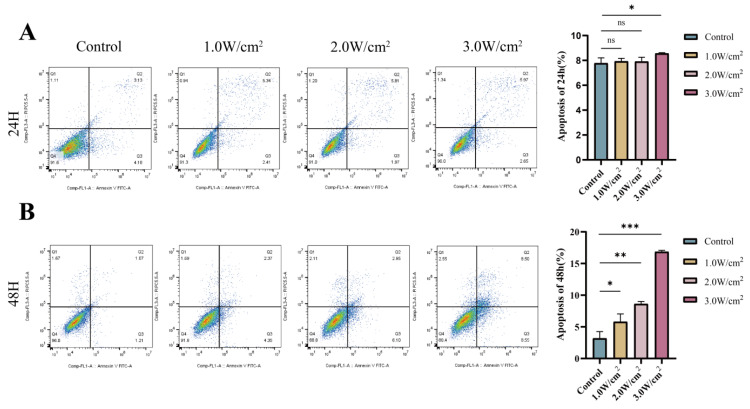
Ultrasound parameter screening result. (A and B) Changes in apoptosis rate of pancreatic cancer cells cultured continuously for 24 or 48 hours after different ultrasound intensities combined with MBs action on pancreatic cancer cells. *p < 0.05 indicates a statistically significant difference.

After 24 hours of continuous culture following UTMD treatment, we observed that the total apoptosis rate did not change significantly at ultrasound intensities of 1.0 W/cm^2^ and 2.0 W/cm^2^, but was significantly elevated at 3.0 W/cm^2^ (compared to the Control group: Control: 7.78 ± 0.42%; 1.0 W/cm^2^: 7.93 ± 0.23%; 2.0 W/cm^2^: 7.91 ± 0.34%; 3.0 W/cm^2^: 8.56 ± 0.06%, Figure 2A). Similarly, after 48 hours of continuous culture following UTMD treatment, the total cell apoptosis rate was not significantly increased at ultrasound intensities of 1.0 W/cm^2^ and 2.0 W/cm^2^, but was significantly higher at 3.0 W/cm^2^ (compared to the Control group: Control = 3.18 ± 1.08%; 1.0 W/cm^2^ = 5.81 ± 1.24%; 2.0 W/cm^2^ = 8.62 ± 0.40%; 3.0 W/cm^2^ = 16.86 ± 0.21%, Figure 2B). Based on the above results, at an ultrasound intensity of 3.0 W/cm^2^, the apoptosis rate was the highest for both 24 H and 48 H of continuous incubation after UTMD treatment compared with the control group. Based on this result, we selected an ultrasound intensity of 3.0 W/cm^2^ for subsequent experiments.

### 3.2. UTMD-induced apoptosis in pancreatic cancer cells

**Figure 3:**
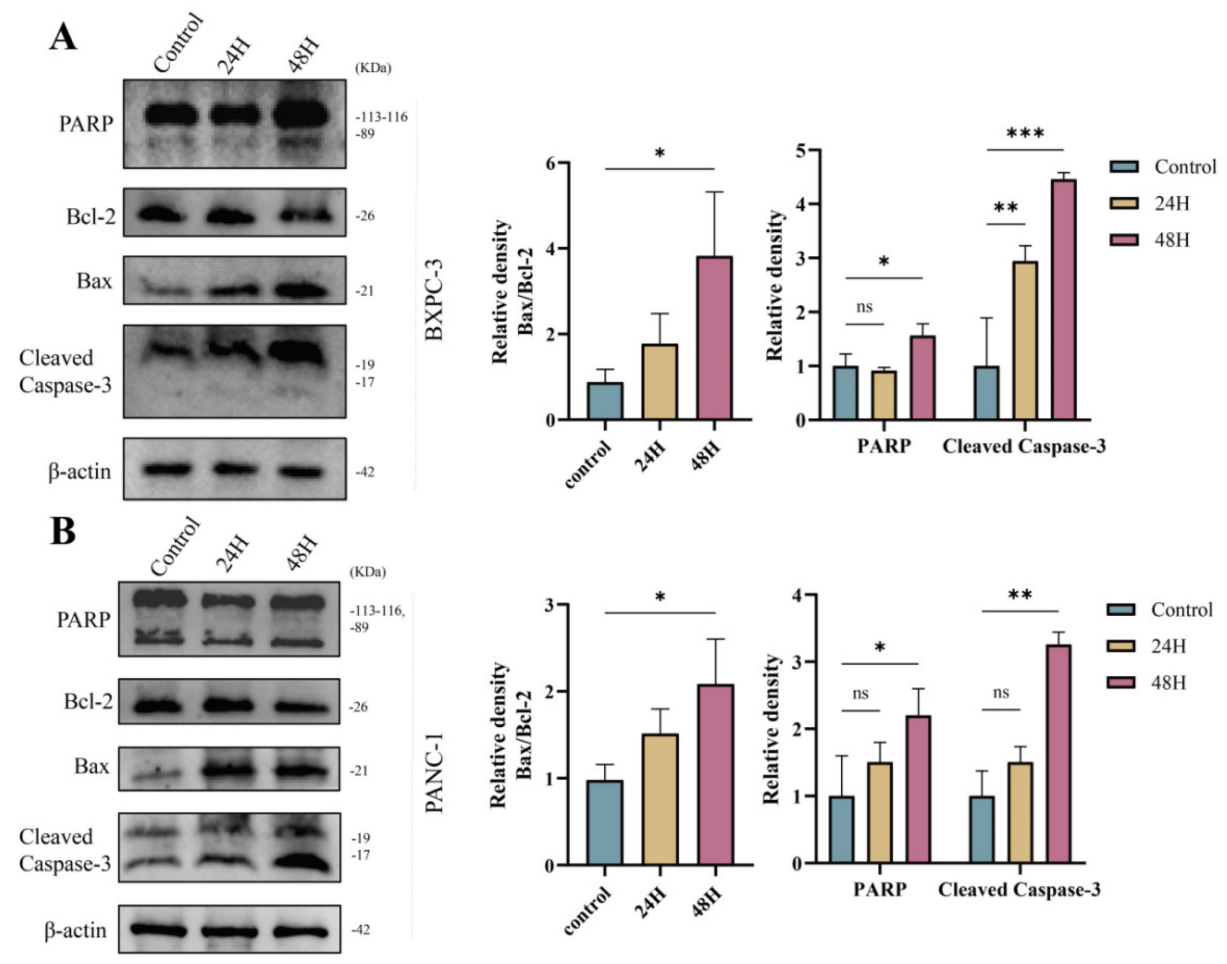
UTMD-induced apoptosis in pancreatic cancer cells. (A and B) Western blot analysis was used to detect the expression of apoptosis marker proteins PARP, Bax/Bcl-2, and Cleaved Caspase-3 in BXPC-3 and PANC-1 pancreatic cancer cells. *p < 0.05 indicates a statistically significant difference.

Data from the western blot analysis showed a significant increase in Cleaved Caspase-3, Bax, and Cleaved-PARP expression, along with a significant decrease in Bcl-2, in the UTMD 48 H group compared to the control and UTMD 24 H groups (Figures 3A and 3B). This is consistent with the flow cytometry analysis, which demonstrated that UTMD enhances apoptosis in pancreatic cancer cells. These results suggest that UTMD can induce apoptosis in pancreatic cancer cells.

### 3.3. UTMD-induced autophagy in pancreatic cancer cells

**Figure 4:**
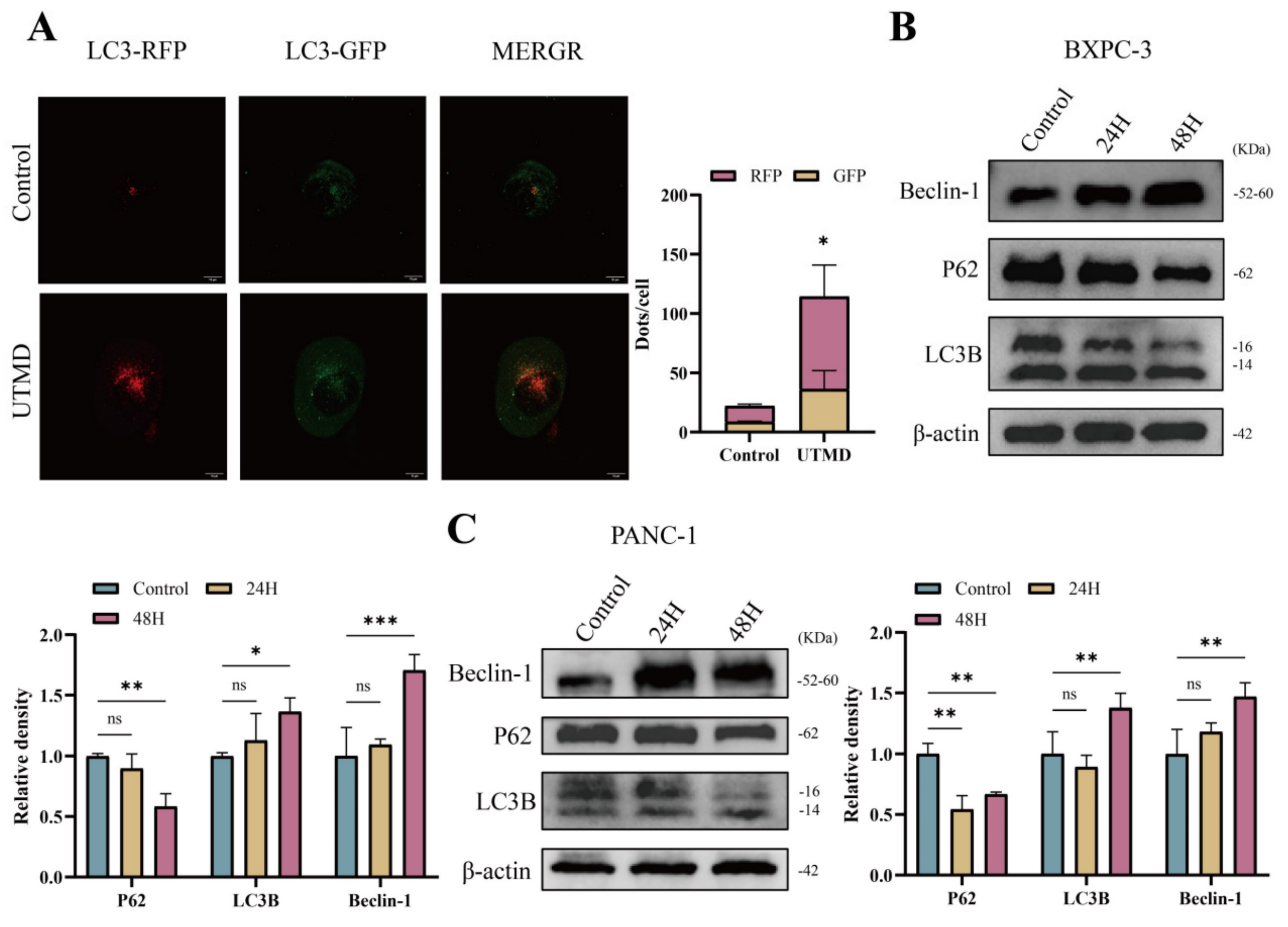
UTMD-induced autophagy in pancreatic cancer cells. (A) Representative images of fluorescent LC3 puncta are shown; scale bar: 10 µm. (B and C) Western blot analysis was used to detect the expression levels of autophagy-related proteins Beclin-1, P62, and LC3B in BXPC-3 and PANC-1 pancreatic cancer cells. *p < 0.05 indicates a statistically significant difference.

In an acidic environment, green fluorescent protein (GFP) is easily degraded, while red fluorescent protein (mCherry) remains relatively stable. Therefore, Ad-mCherry-GFP-LC3 transfection results revealed a significant increase in the number of red/yellow (mCherry merged with green) dots in the UTMD group compared to the Control group (57.2 ± 29.5 vs. 11.0 ± 3.3, P < 0.05), suggesting that UTMD treatment significantly increased the number of autophagosomes in pancreatic cancer cells and that autophagic flux remained unobstructed (Figure 4A). Western blot analysis showed a significant increase in the expression of LC3B-II/I and Beclin-1 in the UTMD 48 H group, while the expression of P62 was decreased compared to the control and UTMD 24 H groups (Figures 4B and 4C). Based on this result, we selected pancreatic cancer cells that were continuously cultured for 48 hours after UTMD treatment for subsequent experiments. Overall, these results suggest that UTMD induces autophagy in pancreatic cancer cells.

### 3.4. Inhibition of UTMD-induced autophagy promotes apoptosis in pancreatic cancer cells

**Figure 5:**
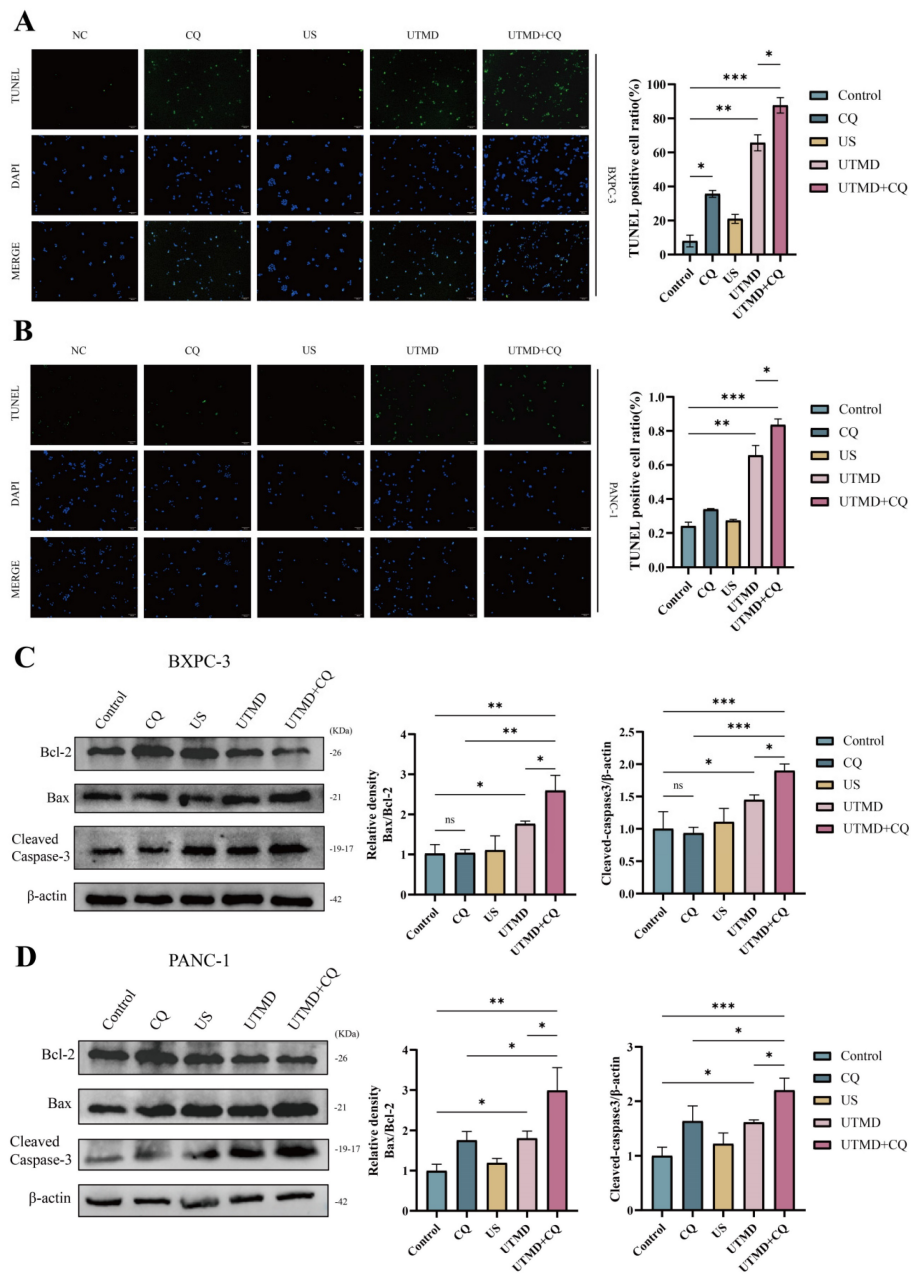
Inhibition of UTMD-Induced Autophagy Promotes Apoptosis in Pancreatic Cancer Cells. (A and B) TUNEL staining was performed to assess apoptosis in BXPC-3 and PANC-1 pancreatic cancer cells; scale bar: 100 µm. (C and D) Western blot analysis was used to detect the expression levels of apoptosis-related proteins Bax/Bcl-2 and Cleaved Caspase-3 in BXPC-3 and PANC-1 pancreatic cancer cells. *p < 0.05 indicates a statistically significant difference.

To investigate the effect of inhibition of autophagy on apoptosis in pancreatic cancer cells, apoptosis was assessed using the TUNEL assay kit. Compared to the control group, the UTMD group showed an increase in apoptosis, while the CQ and US groups did not show significant changes. Additionally, a further increase in apoptosis was observed in the UTMD + CQ group compared to the UTMD group (Figures 5A and 5B). Western blot analysis showed an increase in the expression of Cleaved Caspase-3 and Bax, along with a decrease in Bcl-2 expression, in the UTMD group compared to the control, CQ, and US groups, with a more pronounced trend in the UTMD + CQ group compared to the UTMD group (Figures 5C and 5D). These results suggest that the autophagy inhibitor CQ can enhance UTMD-induced apoptosis in pancreatic cancer cells.

### 3.5. Inhibition of UTMD-induced cell apoptosis does not affect autophagy in pancreatic cancer cells

**Figure 6:**
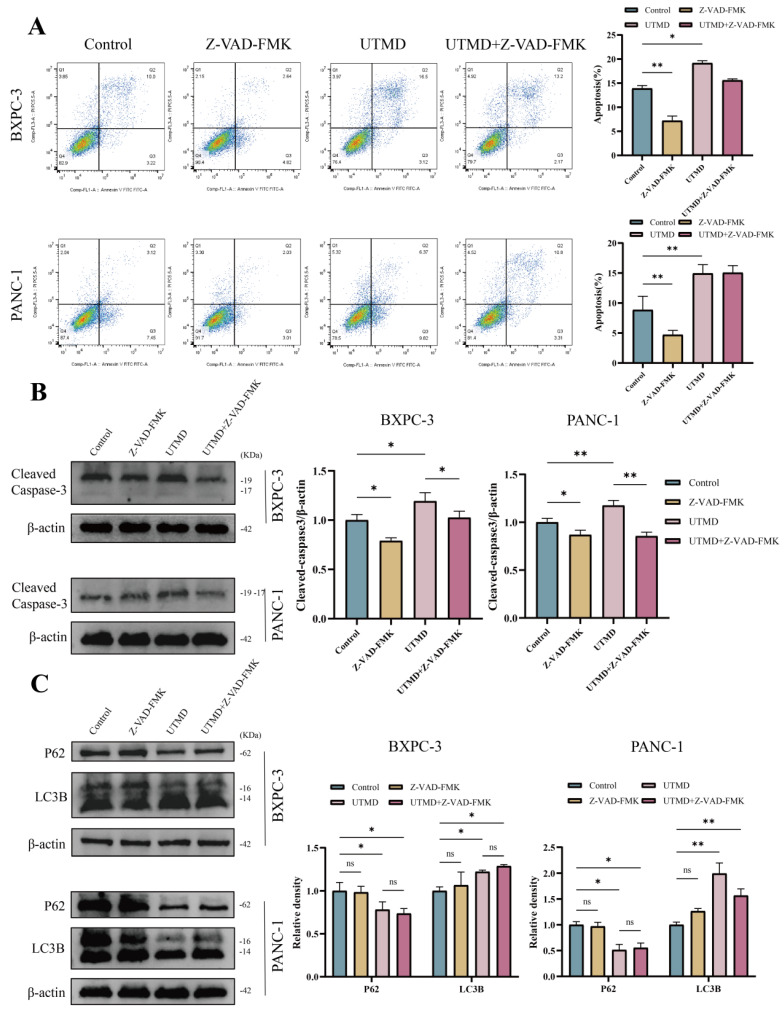
Inhibition of UTMD-induced cell apoptosis did not affect autophagy in pancreatic cancer cells. (A) Flow cytometry was performed to measure the apoptosis rate in BXPC-3 and PANC-1 pancreatic cancer cells. (B) Western blot analysis was used to detect the expression levels of the apoptosis-related protein Cleaved Caspase-3 in BXPC-3 and PANC-1 pancreatic cancer cells. (C) Western blot analysis was used to detect the expression levels of autophagy-related proteins P62 and LC3B in BXPC-3 and PANC-1 pancreatic cancer cells. *p < 0.05 indicates a statistically significant difference.

VAD-FMK is a pan-caspase inhibitor. To determine whether Z-VAD-FMK inhibits apoptosis, the total apoptosis rate was assessed using flow cytometry. The total apoptosis rate was significantly reduced in the Z-VAD-FMK group compared to the control group, and the rate in the UTMD group was consistent with findings from previous experiments (BXPC-3: Control = 13.91 ± 0.60%; Z-VAD-FMK = 7.20 ± 0.98%; UTMD = 19.18 ± 0.48%; UTMD + Z-VAD-FMK = 15.60 ± 0.28%; PANC-1: Control = 8.86 ± 2.27%; Z-VAD-FMK = 4.71 ± 0.72%; UTMD = 14.94 ± 1.46%; UTMD + Z-VAD-FMK = 15.05 ± 1.19%, Figure 6A). Western blot results were consistent with the flow cytometry findings, showing that Cleaved Caspase-3 expression was significantly decreased in the Z-VAD-FMK group and significantly elevated in the UTMD group compared to the Control group (Figure 6B). We found that the expression of autophagy markers was not significantly altered between the Control group and the Z-VAD-FMK group after apoptosis was inhibited by Z-VAD-FMK. LC3B-II/I expression increased, and P62 expression decreased in the UTMD and UTMD + Z-VAD-FMK groups, with no significant differences in LC3B-II/I and P62 expression between these two groups (Figure 6C). In summary, the autophagy inhibitor enhanced UTMD-induced apoptosis in pancreatic cancer cells, while the apoptosis inhibitor did not affect UTMD-induced autophagy.

### 3.6. Inhibition of autophagy enhanced the therapeutic effect of UTMD* in vivo*

**Figure 7:**
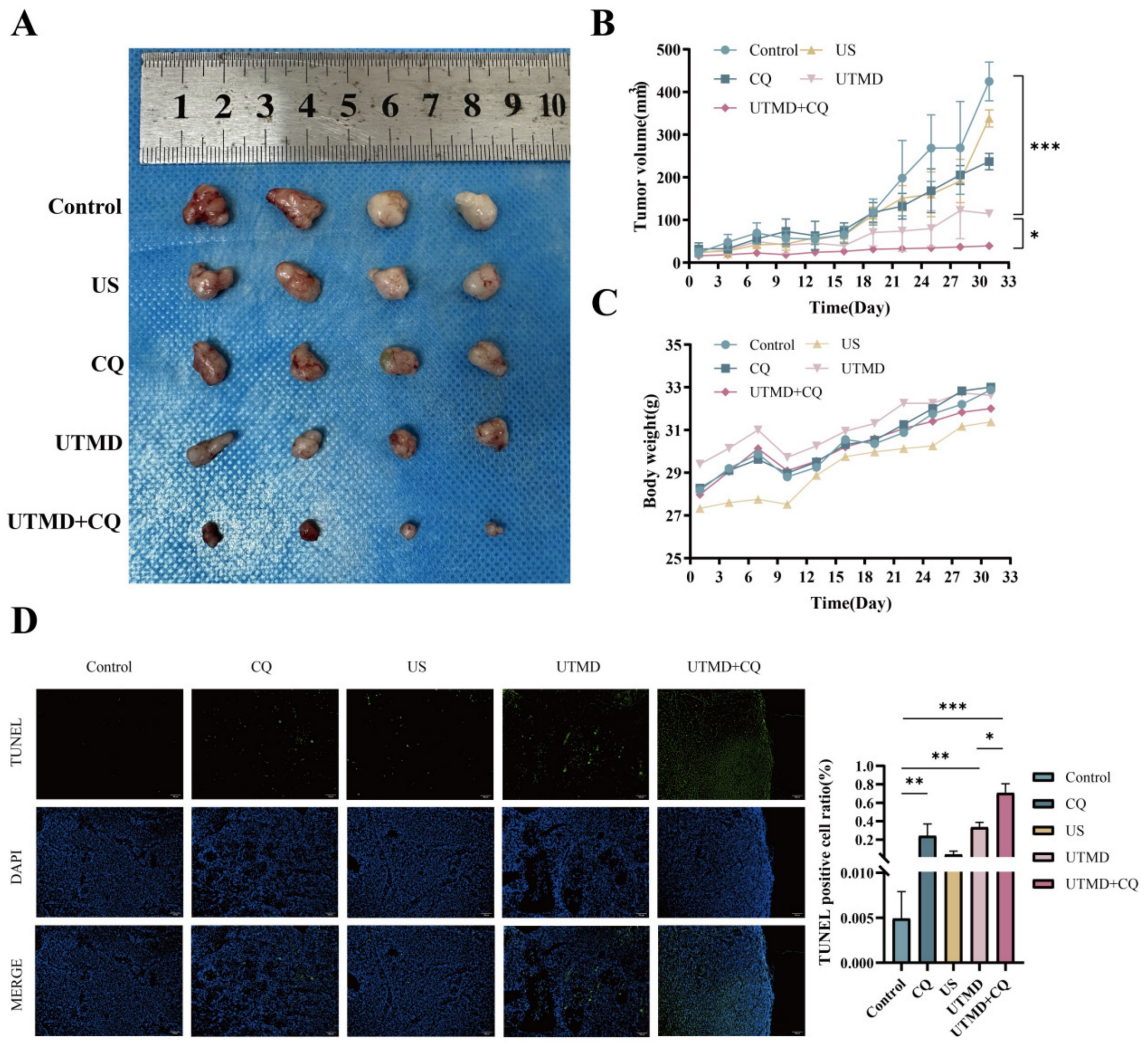
Inhibition of autophagy enhances the therapeutic effect of UTMD *in vivo*. (A-C) Pancreatic tumor xenografts were established in NU/NU nude mice. Equal amounts of chloroquine (CQ, 60 mg/kg) or 0.9% normal saline were injected intraperitoneally every three days; except for the control and CQ groups, the other three groups were treated with the previously identified ultrasound intensity for 5 minutes. Tumor size and weight of nude mice were also recorded every three days. Tumor images (A), tumor size (B), and nude mouse weight (C) are presented. (D) TUNEL staining was performed to assess tumor cell apoptosis; scale bar: 100 µm. *p < 0.05 indicates a statistically significant difference.

To evaluate the therapeutic potential of UTMD *in vivo* after autophagy inhibition, we introduced BXPC-3 pancreatic cancer cells into the axillary region of Nu/Nu nude mice, thereby establishing a xenograft model. Remarkably, in the UTMD + CQ-treated mice, a significant inhibition of xenograft tumor growth was observed, as evidenced by a reduction in tumor volume (Figures 7A-7C). TUNEL staining was used to confirm apoptosis in the resected tumor tissues. In the UTMD group, TUNEL staining showed increased green fluorescence intensity compared to the Control, CQ, and US groups, with this trend being more pronounced in the UTMD + CQ group compared to the UTMD group (Figure 7D). These combined results demonstrate that autophagy inhibition enhanced the therapeutic effect of UTMD *in vivo*.

## 4. Discussion

Pancreatic cancer poses a major threat to human health, with limited treatment options available 18. Currently, UTMD is a promising method for improving anti-tumor efficacy while minimizing side effects on healthy tissues 19. Several studies have shown that UTMD modulates macrophage polarization and vessel normalization to inhibit pancreatic cancer growth and metastasis 20, and its down-regulation of autophagy contributes to increased chemosensitivity in ovarian cancer 21. In the present study, we evaluated the effects of UTMD treatment on apoptosis and autophagy in pancreatic cancer cells and found that UTMD induced both apoptosis and autophagy in these cells (Figures 2 and 3). Further experiments demonstrated that inhibiting autophagy promoted UTMD-induced apoptosis, while apoptosis did not affect UTMD-induced autophagy in pancreatic cancer cells (Figures 4-6).

MBs typically enhance US-induced cavitation 22. In this study, we used MBs and US to treat pancreatic cancer cells and found that the apoptosis rate of pancreatic cancer cells significantly increased after 48 hours of continuous culture following UTMD treatment, compared to the control and UTMD 24 H groups. This was accompanied by enhanced expression of Cleaved Caspase-3 and Bax (pro-apoptotic factors) and decreased expression of Bcl-2 (a key anti-apoptotic factor). These findings are consistent with those of Song *et al.*, who also observed that ultrasound combined with MBs induced apoptosis in pancreatic cancer cells 23. Ultrasound treatment activates Piezo1 and increases its expression 23, 24. Increased intracellular Ca2+ signaling triggers autophagy through ERK activation 25. Yue *et al.* indicated that the upregulation of Piezo1 after ischemic injury leads to Ca2+ influx, thereby promoting cell apoptosis and autophagy 26. Our experiments also demonstrated that UTMD treatment promoted autophagy in pancreatic cancer cells, evidenced by the formation of autophagosomes, a significant increase in LC3B-II/I and Beclin1 expression, and a decrease in P62 expression. Thus, UTMD treatment induces both apoptosis and autophagy in pancreatic cancer cells. UTMD-induced apoptosis and autophagy may be mediated through Piezo1, which we will explore further in our future work. In addition, Western blot analysis revealed a significant increase in the expression of autophagy-related proteins in pancreatic cancer cells cultured for 48 hours after UTMD treatment. This result is similar to the expression of apoptosis-related proteins following UTMD treatment. Studies have shown that high-intensity stress treatment induces mitochondrial damage by reducing mitochondrial membrane potential and oxygen consumption rate in a time-dependent manner 27. Moreover, under excessive mechanical stress, Piezo1 is significantly upregulated in a time-dependent manner, suggesting that Piezo1 expression is closely associated with cell apoptosis 27. Low-intensity pulsed ultrasound (LIPUS) treatment increases the expression of Piezo1 24. Autophagy and apoptosis induced by UTMD are closely associated with Piezo1. The time-dependent expression of Piezo1 following ultrasound treatment is key to reaching peak levels of apoptosis and autophagy-related proteins. Our experiments showed that Piezo1 expression peaked at 48 hours and did not significantly increase afterward (Figure S1). This information provides further clues about the relationship between Piezo1 and UTMD-induced autophagy and apoptosis, which we will explore in our future work.

In tumor therapy, autophagy functions as a double-edged sword. Some studies have shown that autophagy can increase tumor cell tolerance to stress and support their survival in unfavorable environments 28. However, it has also been observed that autophagy can inhibit tumor growth and metastasis at different stages of tumorigenesis and development 29. If autophagy inhibits pancreatic cancer cell apoptosis, the efficiency of UTMD in killing cancer cells can be enhanced by inhibiting autophagy. Conversely, if autophagy promotes pancreatic cancer cell apoptosis, the efficiency of UTMD in killing cancer cells can be further enhanced by promoting autophagy. Therefore, the role of autophagy in pancreatic cancer cell apoptosis is crucial for the clinical application of UTMD in pancreatic cancer treatment. *In vitro* experiments confirmed that CQ treatment significantly enhanced UTMD-induced apoptosis, while Z-VAD-FMK treatment inhibited it. CQ treatment inhibited UTMD-induced autophagy, while Z-VAD-FMK treatment had no effect on autophagy. *In vivo* experiments also confirmed that inhibiting UTMD-induced autophagy enhanced the therapeutic effect of UTMD on pancreatic cancer, indicating that autophagy acts as a protective mechanism in UTMD-treated pancreatic cancer cells. Yes-associated protein (YAP) is a mechanotransducer that senses external mechanical stimulation to cells and converts it into cell-specific transcriptional programs 30, 31. Jian *et al.*'s study showed that LIPUS promotes autophagy and reduces periodontal ligament cell apoptosis by upregulating the expression and nuclear translocation of YAP 32. Piezo1 senses mechanical stimuli, leading to increased Src phosphorylation, which subsequently activates YAP 33. These infomation suggest that autophagy resistance to UTMD-induced apoptosis may be mediated through the Piezo1-YAP pathway. We will explore this further in our future work.

This study confirms our main findings in *in vitro* and *in vivo* models with some limitations. First, the animal models used in the study cannot fully mimic the complex pathological features of human pancreatic cancer, and the sample size and experimental conditions may also limit the generalizability of the results. Additionally, we were unable to fully elucidate the specific molecular mechanisms linking autophagy and UTMD-induced apoptosis. Further studies should validate these results in a broader range of models with larger sample sizes and further investigate the underlying molecular mechanisms involved.

## Supplementary Material

Supplementary figure.

## References

[1] Siegel RL, Giaquinto AN, Jemal A (2024). Cancer statistics, 2024. CA Cancer J Clin.

[2] Bray F, Laversanne M, Sung H, Ferlay J, Siegel RL, Soerjomataram I (2024). Global cancer statistics 2022: GLOBOCAN estimates of incidence and mortality worldwide for 36 cancers in 185 countries. CA Cancer J Clin.

[3] Neoptolemos JP, Kleeff J, Michl P, Costello E, Greenhalf W, Palmer DH (2018). Therapeutic developments in pancreatic cancer: current and future perspectives. Nat Rev Gastroenterol Hepatol.

[4] Rehman M, Khaled A, Noel M (2022). Cytotoxic Chemotherapy in Advanced Pancreatic Cancer. Hematol Oncol Clin North Am.

[5] Strobel O, Neoptolemos J, Jäger D, Büchler MW (2019). Optimizing the outcomes of pancreatic cancer surgery. Nat Rev Clin Oncol.

[6] Vornhülz M, Anton S, Eross B, Szakács Z, Hegyi P, Regel I (2022). Role of stereotactic body radiation in the enhancement of the quality of life in locally advanced pancreatic adenocarcinoma: a systematic review. Radiat Oncol.

[7] Hu Y, Wei J, Shen Y, Chen S, Chen X (2023). Barrier-breaking effects of ultrasonic cavitation for drug delivery and biomarker release. Ultrason Sonochem.

[8] Cao J, Hu C, Zhou H, Qiu F, Chen J, Zhang J (2021). Microbubble-Mediated Cavitation Promotes Apoptosis and Suppresses Invasion in AsPC-1 Cells. Ultrasound Med Biol.

[9] Huang P, You X, Pan M, Li S, Zhang Y, Zhao Y (2013). A novel therapeutic strategy using ultrasound mediated microbubbles destruction to treat colon cancer in a mouse model. Cancer Lett.

[10] Li Y, Wang P, Chen X, Hu J, Liu Y, Wang X (2016). Activation of microbubbles by low-intensity pulsed ultrasound enhances the cytotoxicity of curcumin involving apoptosis induction and cell motility inhibition in human breast cancer MDA-MB-231 cells. Ultrason Sonochem.

[11] Russell RC, Guan KL (2022). The multifaceted role of autophagy in cancer. Embo j.

[12] He L, Zhang J, Zhao J, Ma N, Kim SW, Qiao S (2018). Autophagy: The Last Defense against Cellular Nutritional Stress. Adv Nutr.

[13] He Y, Dong XH, Zhu Q, Xu YL, Chen ML, Liu Z (2022). Ultrasound-triggered microbubble destruction enhances the radiosensitivity of glioblastoma by inhibiting PGRMC1-mediated autophagy in vitro and in vivo. Mil Med Res.

[14] Wang YU, Chen YN, Zhang W, Yang YU, Shen E, Hu B (2015). Upregulation of Beclin-1 expression in DU-145 cells following low-frequency ultrasound irradiation combined with microbubbles. Oncol Lett.

[15] Wu Y, Liu X, Qin Z, Hu L, Wang X (2018). Low-frequency ultrasound enhances chemotherapy sensitivity and induces autophagy in PTX-resistant PC-3 cells via the endoplasmic reticulum stress-mediated PI3K/Akt/mTOR signaling pathway. Onco Targets Ther.

[16] Chen R, Zou J, Zhong X, Li J, Kang R, Tang D (2024). HMGB1 in the interplay between autophagy and apoptosis in cancer. Cancer Lett.

[17] Pan Y, Yoon S, Sun J, Huang Z, Lee C, Allen M (2018). Mechanogenetics for the remote and noninvasive control of cancer immunotherapy. Proc Natl Acad Sci U S A.

[18] Hosein AN, Brekken RA, Maitra A (2020). Pancreatic cancer stroma: an update on therapeutic targeting strategies. Nat Rev Gastroenterol Hepatol.

[19] Liu S, Zhang Y, Liu Y, Wang W, Gao S, Yuan W (2023). Ultrasound-targeted microbubble destruction remodels tumour microenvironment to improve immunotherapeutic effect. Br J Cancer.

[20] Lin L, Du Y, Hao J, Wu R, Du L (2023). UTMD inhibits pancreatic cancer growth and metastasis by inducing macrophage polarization and vessel normalization. Biomed Pharmacother.

[21] Fan G, Qin J, Fu X, Si X, Li L, Yang K (2022). Low-Intensity Focused Ultrasound Targeted Microbubble Destruction Enhanced Paclitaxel Sensitivity by Decreasing Autophagy in Paclitaxel-Resistant Ovarian Cancer. Front Oncol.

[22] Chowdhury SM, Abou-Elkacem L, Lee T, Dahl J, Lutz AM (2020). Ultrasound and microbubble mediated therapeutic delivery: Underlying mechanisms and future outlook. J Control Release.

[23] Song Y, Chen J, Zhang C, Xin L, Li Q, Liu Y (2022). Mechanosensitive channel Piezo1 induces cell apoptosis in pancreatic cancer by ultrasound with microbubbles. iScience.

[24] Gao Q, Cooper PR, Walmsley AD, Scheven BA (2017). Role of Piezo Channels in Ultrasound-stimulated Dental Stem Cells. J Endod.

[25] Bootman MD, Chehab T, Bultynck G, Parys JB, Rietdorf K (2018). The regulation of autophagy by calcium signals: Do we have a consensus?. Cell Calcium.

[26] Yue Y, Chen P, Ren C (2024). Piezo1 Modulates Neuronal Autophagy and Apoptosis in Cerebral Ischemia-Reperfusion Injury Through the AMPK-mTOR Signaling Pathway. Neurochem Res.

[27] Shi S, Kang XJ, Zhou Z, He ZM, Zheng S, He SS (2022). Excessive mechanical stress-induced intervertebral disc degeneration is related to Piezo1 overexpression triggering the imbalance of autophagy/apoptosis in human nucleus pulpous. Arthritis Res Ther.

[28] Chen G, Gao C, Jiang S, Cai Q, Li R, Sun Q (2024). Fusobacterium nucleatum outer membrane vesicles activate autophagy to promote oral cancer metastasis. J Adv Res.

[29] Chen YM, Xu W, Liu Y, Zhang JH, Yang YY, Wang ZW (2023). Anomanolide C suppresses tumor progression and metastasis by ubiquitinating GPX4-driven autophagy-dependent ferroptosis in triple negative breast cancer. Int J Biol Sci.

[30] Mannion AJ, Zhao H, Zhang Y, von Wright Y, Bergman O, Roy J (2024). Regulation of YAP Promotor Accessibility in Endothelial Mechanotransduction. Arterioscler Thromb Vasc Biol.

[31] Panciera T, Azzolin L, Cordenonsi M, Piccolo S (2017). Mechanobiology of YAP and TAZ in physiology and disease. Nat Rev Mol Cell Biol.

[32] Jian Z, Li Y, Zhang C, Zhong W, Ai D, He Y (2023). Low-Intensity Pulsed Ultrasound Attenuates Periodontal Ligament Cells Apoptosis by Activating Yes-Associated Protein-Regulated Autophagy. Ultrasound Med Biol.

[33] Kim OH, Choi YW, Park JH, Hong SA, Hong M, Chang IH (2022). Fluid shear stress facilitates prostate cancer metastasis through Piezo1-Src-YAP axis. Life Sci.

