# Supplementary material for: Inhibition of autophagy promotes ultrasound‑targeted microbubble destruction-induced apoptosis of pancreatic cancer cells
Source: Int J Med Sci. 2025 Mar 3;22(7):1708–19. doi: 10.7150/ijms.106509 (PMC11905268; doi:10.7150/ijms.106509)
Supplement: Supplementary file 1 — Supplementary figure. [file ijmsv22p1708s1.pdf]

A

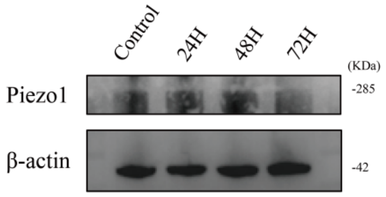

Figure S1. Expression of Piezo1 protein. (A) The levels of Piezo1 protein in pancreatic cancer cells cultured continuously for 24, 48, and 72 hours after treatment with ultrasound combined with MBs.
